# Characterizing Unipolar and Bipolar Depression by Alterations in Inflammatory Mediators and the Prefrontal-Limbic Structural Network

**DOI:** 10.1155/2023/5522658

**Published:** 2023-05-24

**Authors:** Xiaoxia Lei, Juanjuan Ren, Xinyue Teng, Chaoyue Guo, Zenan Wu, Lingfang Yu, Xiaochang Chen, Lirong Fu, Rong Zhang, Dandan Wang, Yan Chen, Yi Zhang, Chen Zhang

**Affiliations:** ^1^Division of Mood Disorders, Shanghai Mental Health Center, Shanghai Jiao Tong University School of Medicine, Shanghai 200030, China; ^2^Shanghai Key Laboratory of Psychotic Disorders, Shanghai Mental Health Center, Shanghai Jiao Tong University School of Medicine, Shanghai, China

## Abstract

**Objective:**

The prefrontal-limbic system is closely associated with emotion processing in both unipolar depression (UD) and bipolar depression (BD). Evidence for this link is derived mostly from task-fMRI studies, with limited support from structural findings. Therefore, this study explores the differences in the emotional circuit in these two disorders on a structural, large-scale network basis, coupled with the highly noted inflammatory and growth factors.

**Methods:**

In this study, 31 BD patients, 37 UD patients, and 61 age-, sex-, and education-matched healthy controls (HCs) underwent diffusion-weighted imaging (DWI) scanning and serum cytokine sampling. The study compared cytokine levels and prefrontal-limbic network alterations among the three groups and explored potential biological and neurobiological markers to distinguish the two disorders using graph theory, network-based statistics (NBS), and logistic regression.

**Results:**

Compared to BD patients, UD patients showed greater s-100*β* protein levels, higher efficiency of the right amygdala, and significantly elevated prefrontal-cingulate-amygdala subnetwork intensity. Importantly, the altered prefrontal-cingulate-amygdala subnetwork, nodal efficiency of the right amygdala, IL-8, IL-17, and s-100*β* levels were risk factors for the diagnosis of UD, whereas anxiety symptoms tended to closely correlate with BD. Moreover, binary logistic regression manifested these factors achieved an area under the curve (AUC) of the receiver operating characteristics (ROC) of 0.949, with 0.875 sensitivity and 0.938 specificity in UD vs. BD classification.

**Conclusions:**

These findings narrow the gap in the structural network of emotional circuits in bipolar and unipolar depression, pointing to distinct emotion-processing mechanisms in both disorders.

## 1. Introduction

Based solely on symptoms for diagnosis, bipolar depression (BD) is highly likely to be misdiagnosed as unipolar depression (UD), especially in the context of an unclear history of manic or hypomanic episodes, with a high misdiagnosis rate of 78.5% [1]. Giving BD patients inappropriate antidepressant therapy may lead to worse outcomes, such as nonresponse, rapid loss of drug efficacy, or resistance to treatment with different antidepressants [2]. Therefore, it is necessary to explore the differences between BD and UD from multiple perspectives. This study is aimed at identifying neuroimaging and immunological markers that can help differentiate between BD and UD, thereby helping to prevent inappropriate treatment and improve outcomes for patients.

As mood disorders, bipolar and unipolar depression are characterized by abnormal emotion processing. Following the emergence of numerous emotion-related task functional magnetic resonance imaging (fMRI) studies, the prefrontal-limbic system was believed to be dysfunctional during emotion processing and regulation in major depressive disorder and bipolar disorder [3–5]. How does the emotional circuit differ between the two depressive subgroups? A growing number of neuroimaging studies reported functional and structural abnormalities in prefrontal-limbic areas between bipolar and unipolar depression [6–13]. Comprehensive findings of task-fMRI studies reported aberrant neural activation patterns to emotional stimuli, particularly in the medial prefrontal gyrus, amygdala, insula, and anterior cingulate gyrus in UD patients compared to BD patients [6, 10, 14]. Similarly, differences in grey matter volume (GMV) and white matter integrity have also been observed within the emotional circuit in both subtypes of depression, such as significantly decreased white matter integrity in the corpus callosum and posterior cingulum [9] and reduced GMV in the hippocampal formation, amygdala, prefrontal cortex, and parietal regions in BD patients relative to those with UD [7, 8].

Even though previous studies yielded substantial and abundant findings to elucidate the potential neural mechanisms of the emotional circuit in unipolar and bipolar depression, the results are often challenging to compare and integrate, as described in the latest Science review [15]. This review also suggested that atlas-based workflows can facilitate brain-wide analyses of neural network organization and advance our understanding of brain function and changes [15]. Relative to rich task-targeted fMRI studies on emotional processing, rare studies explored differences in the prefrontal-limbic circuit at a brain-wide level in unipolar and bipolar depression. Thus, the prime goal in this study was to investigate the differences in the emotional circuit on a white matter structural network basis in the two disorders using atlas-based network analyses.

Additionally, immunological disturbance, correlated with depressive episodes and involved in hypothalamic-pituitary-adrenal axis and vegetative disturbances [16–18], is considered to play an unmissable role in depression and bipolar disorder [19–21]. Few researchers attempted to find an immunological basis to differentiate BD from UD [22–25]. They found decreased cytokine levels, such as neuropeptide Y [26], leptin [27], and brain-derived neurotrophic factor (BDNF) [22] in BD relative to UD, as well as increased levels of kynurenine, kynurenine/tryptophan ratio, and most interleukins (IL) [25]. Others also reported no differences in the levels of orexin A, ghrelin [26], BDNF [23], C-reactive protein (CRP) [28], and IL-6, tumor necrosis factor-*α* (TNF-*α*) [18] between the two affective disorders. Consequently, based on the limited evidence, we are interested in exploring the discrepancy in inflammatory mediators and growth factors between bipolar and unipolar depression.

Overall, this study is aimed at investigating the differences in the emotional circuit on a structural basis of white matter, further comparing the levels of inflammatory and growth factors, and finally identifying potential cytokines and neuroimage markers to differentiate the two affective disorders.

## 2. Materials and Methods

### 2.1. Subjects and Clinical Assessments

This study is cross-sectional and case-control research. The Ethics Committee of the Shanghai Mental Health Center of the School of Medicine of Shanghai Jiao Tong University has approved the research protocol. All subjects were informed of the purpose of the study and provided documented informed consent. All investigations were conducted in strict adherence to the Declaration of Helsinki. The study has been registered on ClinicalTrials.gov (NCT03790085).

Patients were recruited into this study progressively at the Shanghai Mental Health Center outpatient department between August 2019 and December 2020. Ultimately, 31 bipolar-depressed and 37 unipolar-depressed adult patients participated in the study. Patients were diagnosed by the consensus of two senior psychiatrists with the following inclusion criteria: (1) meeting the diagnostic criteria for major depression and bipolar depression based on the Structured Clinical Interview for DSM-IV (SCID), (2) aged between 18 and 45, and (3) having a total score of more than 17 on the Hamilton Depression Rating Scale (HAM-D) 17 items [29]. The exclusion criteria are as follows: (1) meeting the diagnosis or history of other axes I and II disorders such as schizophrenia, substance-induced mood disorder, personality disorders, and obsessive-compulsive disorder and (2) not suitable for MRI. Among the patients, 83.8% of those with UD and 83.9% of those with BD were first-episode unmedicated patients, with some having received antidepressants (duloxetine, milnacipran, escitalopram, and sertraline), mood stabilizers (lithium, valproic acid, lamotrigine, and quetiapine), and antipsychotics (sulpiride and olanzapine) in the past two weeks. Two BD patients had comorbid anxiety disorders, and three UD patients had somatic diseases. The three patients with digestive system diseases were not taking medication at the time of scanning. During the same period, 61 healthy volunteers matched for age, sex, education, handedness, marital status, economic status, and smoking status were recruited from local communities and schools through offline posters and advertisements. All volunteers were interviewed using SCID to exclude any history of neuropsychiatric illness. The HAM-D and Hamilton Anxiety Rating Scale (HAM-A) [30] were used to assess the severity of depressive and anxiety symptoms in BD and UD patients.

### 2.2. Determination of Fasting Plasma Cytokine Levels

Peripheral blood (10 ml) was sampled from 34 UD patients, 17 BD patients, and 55 HCs in the morning; then, the blood samples were immediately centrifuged (3000 × g, 15 min) at 4°C; plasma samples were stored at -80°C until they were used for assays. We used enzyme-linked immunosorbent assays (ELISA) kits (R&D Systems, Minneapolis, MN, USA) to measure the plasma levels of cytokines. Inflammatory mediators evaluated in this study included CRP, interleukins (IL_1*β*, IL_2, IL_4, IL_6, IL_8, IL_10, IL_12, and IL_17), interferon-gamma (IFN-*γ*), TNF_*α*, and s-100*β* protein. Neuron and glial cell-derived growth factors were also sampled, including BDNF and glial-derived neurotrophic factor (GDNF).

### 2.3. MRI Acquisition and Preprocessing

All participants were scanned with a 3.0-Tesla and 64-channel head coil Siemens Magnetom Prisma system (Siemens Healthcare, Erlangen, Germany). They were required to remain still during the scanning. Foam pads and earplugs were used to minimize noise exposure and head movements. T1-weighted brain structural images were obtained using the magnetization-prepared rapid acquisition gradient-echo sequence (repetition time = 2000 ms, echo time = 2.32 ms, flip angle = 8°, number of slices = 208, slice thickness = 0.9 mm, acquisition matrix = 256 × 256, and voxel size = 0.9 × 0.9 × 0.9 mm^3^). Subsequently, a single-shot echo-planar imaging sequence was applied for diffusion-weighted imaging (DWI, repetition time = 3500 ms, echo time = 86 ms, flip angle = 90°, number of slices = 92, slice thickness = 1.5 mm, voxel size = 1.5 × 1.5 × 1.5 mm^3^, *b*values = 0, and 1000 s/mm^2^). A qualified radiologist checked the images to exclude brain structural abnormalities. The DWI data were preprocessed based on the procedures implemented in the Diffusion Toolbox of FSL (version 6.0, https://fsl.fmrib.ox.ac.uk/fsl/fslwiki/FDT/UserGuide), including conversion from DICOM to NIFTI, removal of images affected by significant artifacts, brain extraction (BET), distortion correction (EDDY), and fitting of diffusion tensors (DTIFIT).

### 2.4. Construction of the Prefrontal-Limbic Network

#### 2.4.1. Node Definition

In the latest Science review, Leergaard and Bjaalie advised using atlas-based workflows to facilitate brain-wide analyses of neural network organization [15]. The automated anatomical labeling atlas (AAL) defines the prefrontal-limbic system, including 32 cortical and subcortical regions [31]. We therefore applied the atlas to define the nodes of the emotion circuit (see Supplemental Table 1 for detailed definitions and abbreviations of the nodes).

#### 2.4.2. Edge Definition by Fiber Tracking

First, in coupling T1 and fractional anisotropy (FA) images, individual T1-weighted images were first registered to corresponding FA images in the native diffusion space using a linear transformation. The registered T1-FA images were then mapped to the MNI space using a nonlinear transformation. The inverse transformation then warped the AAL nodal masks from the MNI space to the native diffusion space. Next, we performed deterministic fiber tracking to calculate the FA values of white matter fibers between any two nodes to define as edges. Ultimately, we constructed FA-based network matrices consisting of 32 nodes and FA edges. The deterministic fiber tracking was taken in the Pipeline for Analyzing braiN Diffusion imAges (PANDA, version 1.3.1, https://www.nitrc.org/projects/panda/) [32], a MATLAB toolbox based on the FMRIB's diffusion toolbox of the FSL, with default parameters (propagation algorithm = FACT, angle threshold = 45°, and FA threshold = 0.2~1).

#### 2.4.3. Graph Theory Analysis

Network matrices generated from the last step were used to perform graph theory analysis using a graph theoretical network analysis toolbox on MATLAB (GRETNA, version 2.0, https://www.nitrc.org/projects/gretna/) [33]. In the weighted networks with a set of network sparsity from 0.05 to 0.50 with an interval of 0.05 and 100 random networks, we calculated classical global network metrics such as shortest path length (*L*_*p*_), global efficiency (*E*_glob_), local efficiency (*E*_loc_), and nodal properties including node efficiency (Ne) and nodal local efficiency (NLe). Considering the space of the manuscript, a detailed explanation of network properties is shown in the Supplemental materials. False discovery rate (FDR) correction was used to determine the statistical significance of group differences.

#### 2.4.4. Network-Based Statistics (NBS)

To identify enhanced/attenuated subnetwork intensity in the prefrontal-limbic system in bipolar and unipolar depression, we conducted NBS analyses [34] to explore significant between-group differences. In the premise of component *p* and edge *p* both below 0.05, we determined significantly altered network edges using two-sample *t*-tests among the HC, BD, and UD groups, with age and gender as covariates. For estimating the significance of each subnetwork, permutation tests randomly assigned the subjects into HC, BD, and UD groups 10,000 times and generated a null distribution of the network size. The significance of survived subnetworks was then determined by comparing the original network sizes with the null distribution with a threshold *p* < 0.05, corrected for by family-wise error (FWE).

### 2.5. Statistical Analysis

Demographic, scale, and biochemical data were compared among the three groups using analysis of variance (ANOVA) followed by the Bonferroni post hoc test for multiple comparisons, using the Statistical Package for the Social Sciences (SPSS-26; SPSS Inc., Chicago, IL, USA). Pearson's chi-square (*χ*^2^) tests were for categorical variables. We also explored the differences between groups between unipolar and bipolar depression using two independent sample *t*-tests.

Then, using binary logistic regression analyses with the stepwise forward method, we included variables in three aspects to investigate which factors were effective predictors of the two affective disorders, with BD and UD coded as 0 and 1, respectively. These variables included total scores of HAM-A and HAM-D, white matter network properties, and cytokines, including inflammatory mediators and growth factors, with predictors reaching *p* < 0.05 considered significant. The area under the receiver operating characteristics (ROC) curve (AUC) was calculated for the ability to differentiate unipolar depression from bipolar depression by visualizing the false positive rate (1-specificity) versus the true positive rate (sensitivity) of those risk factors. Generally, an AUC of 0.5 indicates no discrimination, 0.7 to 0.8 suggests acceptable discrimination, 0.8 to 0.9 is excellent discrimination, and more than 0.9 is considered outstanding discrimination [35].

## 3. Results

### 3.1. Demographic and Clinical Comparisons

There were no statistically significant differences among the three groups in terms of age, sex, education, income, marriage, and smoking (*F* = 2.54, *p* = 0.08; *χ*^2^ = 1.32, *p* = 0.52; *F* = 0.67, *p* = 0.51; *F* = 3.82, *p* = 0.70; *F* = 0.69, *p* = 0.95; *F* = 0.81, *p* = 0.46). The course of the two affective disorders differed significantly (*t* = 2.42, *p* = 0.02), with a longer course in the BD group. With regard to anxiety and depressive symptoms, both unipolar and bipolar patients exhibited significantly higher total scores of HAM-D (*F* = 301.24, *p* < 0.0001) and HAM-A (*F* = 229.68, *p* < 0.0001) compared to healthy controls, but there were no significant differences between the UD and BD groups. Please see Table 1 for detailed statistics.

### 3.2. Comparison of Plasma Cytokine Levels

The plasma inflammatory mediators and growth factors were compared among the three groups and between patient subgroups. No significant differences in all biological indicators were found among the three groups. However, the two-sample *t*-test showed slightly higher levels of s-100*β* protein in the UD group (*t* = 2.02, *p* = 0.049) compared to the BD group. See Supplemental Table 2 for detailed statistics.

### 3.3. Alterations in the Graph-Theoretical Properties of the Prefrontal-Limbic Network

**Figure 1:**
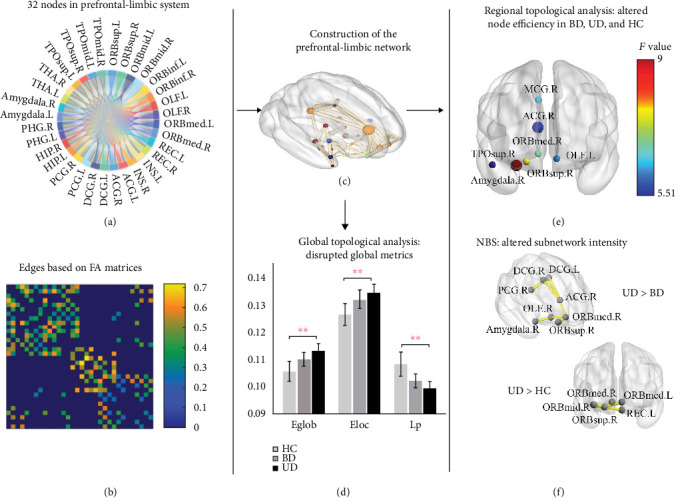
The flowchart of the prefrontal-limbic network construction and the results of significantly altered network nodes and edges. (a) Lists of 32 cortical and subcortical areas as nodes related to the emotion processing circuit. (b) The edges of the network were derived from the FA values between any two nodes using deterministic fiber tracking. (c) A weighted white matter network in the prefrontal-limbic system of a healthy participant. (d, e) Altered global and local network properties. From the graph, it was clear that unipolar patients showed increased global and local efficiency but decreased the length of the shortest path relative to healthy controls, suggesting an overused structural network in the emotion circuit particularly in the right amygdala and prefrontal orbital areas. However, BD patients only demonstrated statistically increased nodal efficiency in the right anterior cingulate gyrus. Importantly, the nodal efficiency of the right amygdala reached a dramatic change between UD and BD, which indicated different mechanisms of emotion processing in the two distinct depressions. The color bar in (e) represents the *F* values in the ANOVA tests. (f) Altered subnetworks in NBS analyses. ⁣^∗∗^*p* < 0.01.

At the whole-brain level, only the UD group showed statistical differences compared to HCs, with significantly enhanced *E*_glob_ and *E*_loc_ and decreased *L*_*p*_. At the local-brain level, both patient subgroups showed increased nodal efficiency compared to HCs, with specific regions showing significant differences. In the UD group, the nodal efficiency of several brain regions, including the ORBsup.R, ORBmed.R, OLF.L, ACG.R, TPOsup.R, and Amygdala.R, as well as the nodal local efficiency of MCG.R, was significantly higher compared to HCs. In the BD group, the nodal efficiency of ACG.R was significantly elevated compared to HCs. Notably, there were differences between the two depression subgroups, with the UD group showing significantly higher nodal efficiency of the right amygdala compared to the BD group. For more detailed statistics, please refer to Table 2 and Figure 1.

### 3.4. Altered Subnetwork within the Prefrontal-Limbic System

The NBS analysis identified significant differences in subnetworks within the prefrontal-limbic system among the three groups. Compared with the HCs, patients with unipolar depression exhibited a significantly increased subnetwork intensity containing five nodes and four edges located in the medial prefrontal areas (threshold edge *t* > 2.50, component *p* = 0.042, FWE corrected). Furthermore, compared to the BD group, the UD group also showed a significantly increased subnetwork intensity consisting of eight nodes and seven edges located in the prefrontal-cingulate-amygdala circuit (threshold edge *t* > 2.00, component *p* = 0.018, FWE corrected). No significant results were derived from the contrast of “BD vs. HC.” Additionally, the mean value of the matrix in each subject was extracted to obtain the subnetwork intensity for the following regression analysis. Supplemental Table 3 and Figure 1 provide detailed information on the components of the altered subnetworks.

### 3.5. Predicting Risk Factors and Categorical Diagnosis in Patient Groups

In binary logistic regression, variables were selected using the stepwise forward method. Seven variables reached the Akaike information criterion optimization and showed a significantly better fit than the null model (*χ*^2^ = 37.37, df = 7, *p* < 0.001). Nagelkerke's *R*^2^ (0.713) indicated a moderately strong relationship between predictors and variable grouping. In the final model, 6 out of 7 variables (s-100*β*, IL-8, IL-17, amygdala nodal efficiency, intensity of the prefrontal-cingulate-amygdala subnetwork, and the HAM-A score) were effective in differentiating patients with UD from those with BD. Related odds rates (ORs) with 95% confidence intervals (CIs) are shown in Table 3. The ORs suggested that IL-17, IL-8, s-100*β*, the nodal efficiency of the right amygdala, and the prefrontal-cingulate-amygdala subnetwork intensity were risk factors for UD. On the contrary, the HAM-A score was positively associated with BD, suggesting that the anxiety symptom is a risk factor for BD.

**Figure 2:**
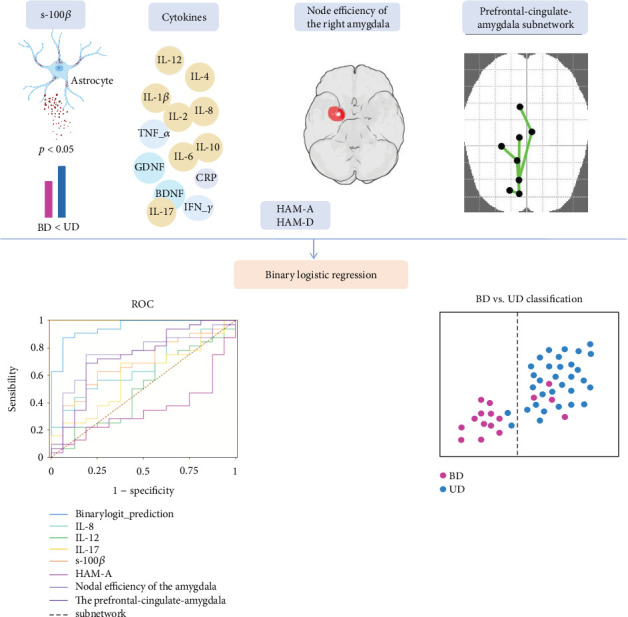
The workflow of prediction and classification. Before the logistic regression analysis, we found slightly higher s-100*β* protein in the UD group than in the BD group, as described in the figure. Secreted by astrocytes, s-100*β* protein is often correlated with many brain injury diseases and mental illnesses. s-100*β*, together with the significantly changed prefrontal-limbic network properties, was enrolled in the binary logistic regression, which also included the total score of HAM-D and HAM-A, CRP, IL_1*β*, IL_2, IL_4, IL_6, IL_8, IL_10, IL_12, IL_17, s-100*β*, IFN_*γ*, TNF_*α*, BDNF, and GDNF. In the stepwise forward method, six variables (s-100*β*, IL-8, IL-17, amygdala nodal efficiency, prefrontal-cingulate-amygdala subnetwork intensity, and HAM-A score) were effective in differentiating patients with UD from those with BD. The AUC value of the BinaryLogit prediction model was 0.949, with a sensitivity of 0.875 and a specificity of 0.938, with an overall prediction accuracy of 87.50%, in which its prediction value was higher than the separate prediction value of each factor.

Of note, prediction success was 94% (30 of 32) for UD and 75% (12 of 16) for BD. The overall prediction accuracy of the model was 87.50%, suggesting that the fit of the model was acceptable. As reported in Table 3, the AUC value of the binary logit prediction model was 0.949, with a sensitivity of 0.875 and a specificity of 0.938, in which its prediction value is higher than the separately predicted value of each factor. See Figure 2 for the workflow.

## 4. Discussion

Combining cytokine and DWI data, we detected differences between groups in inflammatory mediators, growth factors, and network properties in the emotional circuit among the HC, BD, and UD groups, trying to find valuable biomarkers to differentiate BD and UD. We mainly found significantly increased s-100*β* protein levels, nodal efficiency of the right amygdala, and the prefrontal-cingulate-amygdala subnetwork intensity in UD patients, compared with BD patients. Equally important was that logistic regression manifested increased levels of inflammatory mediators (IL-8, IL-17, and s-100*β*), nodal efficiency of the right amygdala nodal network, and the intensity of the prefrontal-cingulate-amygdala subnetwork were risk factors for UD, while the high anxiety symptom was a risk factor for BD. The unipolar vs. bipolar depression classification achieved an acceptable prediction accuracy of 87.50%.

### 4.1. The Key Role of the Amygdala and Its Subnetwork in Distinguishing BD and UD

Emotion processing-related brain regions in the prefrontal-limbic system have an abnormal structural basis in both grey and white matter in unipolar and bipolar depression [7, 8, 11]. As the center of the prefrontal-limbic system, the amygdala experienced significant alterations, with a decrease in GMV [8] but a greater mean diffusion (MD) of white matter [36] in BD compared to UD. Similarly, as a crucial link in the emotional circuit, the cingulate gyrus and its attached white fibers showed discrepancies between the two depression groups, with increased GMV in the left anterior cingulate [8, 11] but decreased FA in the corpus callosum and cingulum [9, 25, 37] in the BD group. Additionally, other altered limbic regions showed UD-BD differences, in which BD patients had thinner GMV in the right caudal middle frontal region, the left inferior parietal, and the right precuneus regions [7], with inconsistent increased GMV in the right hippocampus/parahippocampus [11], or decreased GMV in the bilateral hippocampal formation [8].

Although these data-driven whole-brain analyses have reported a considerable number of findings related to the brain structure, these results are relatively discrete and lack an intrinsic framework to integrate into explaining the emotion-processing mechanism in the two distinct disorders. To address this limitation, we directly investigated the structural network of white matter of the prefrontal-limbic system to explore possible alterations in the emotional circuit. In line with previous studies, we confirmed that the amygdala, the most crucial hub in emotion processing, exhibited statistically significant structural differences between the BD and UD groups [8, 36]. A previous task-fMRI study described that BD patients showed lower amygdala activation than UD patients during the processing of threats, sad, neutral, and happy emotions [10], which is consistent with our finding that the efficiency of the amygdala nodes is decreased in BD patients. Of utmost importance, our findings, which the network-based statistical analysis, further showed a decrease in prefrontal-cingulate-amygdala subnetwork intensity in BD compared to UD and also provide a basis in the brain's structural network for abnormalities in the function of the emotion circuit. Previous studies discovered that BD patients exhibited lower connectivity of the amygdala to the insula and hippocampus for threat and the medial orbitofrontal cortex for happy processing [10], as well as showed decreased activity in the insula and temporal cortex for happy faces and the frontal precentral cortex for fearful faces [6], which was partly consistent with an altered amygdala subnetwork in this study.

In all, by combining previous grey and white matter MRI studies, emotion task-fMRI studies, and our findings, we discovered a structural and functional coupling in the amygdala in BD patients, with decreased GMV, altered white matter, decreased nodal efficiency in the structural network, and lower functional activity in emotion processing, relative to UD patients [8, 10, 36]. Undoubtedly, the amygdala and the prefrontal-cingulate-amygdala subnetwork play different key roles in emotion processing in unipolar and bipolar patients, which can help discriminate between patients with BD and UD.

### 4.2. Significantly Altered s-100*β* Levels in BD and UD

Regarding cytokines, many studies have reported their essential correlations with mental illness, especially in unipolar and bipolar patients [18, 22–25, 28]. Inflammatory cytokines have been reported to be associated with the efficacy of antidepressants and have shown a notable decrease after treatment [38, 39]. Moreover, some cytokines, including IFN-*γ*, TNF-*α*, and IL-6, were involved in the tryptophan (Trp) pathological pathway, which is thought to mediate the effects of immune activation on mood regulation [25]. Similarly, neuronal cell-related growth factors BDNF and GDNF are also considered to play crucial roles in the fluctuations in neurotransmitters in mood disorders [40, 41]. Unlike previous studies, which found BD patients had lower serum BDNF levels [22] and higher levels of most immune/inflammatory analytes, such as IL-1*β*, IL-2, IL-4, IL-6, IL-9, and TNF-a, compared with UD [25], we failed to discover significant differences between BD and UD. However, we found a slightly higher s-100*β* protein in the UD group than in the BD group. s-100*β* is a Ca2+ binding protein secreted by astrocytes, and high levels of extracellular s-100*β* have been detected in brain trauma, ischemia, neurodegenerative, and inflammatory and psychiatric diseases [42–44]. Additionally, high s-100*β* levels at baseline were associated with better treatment response in major depressive disorder [43]. Our finding was the first to find a significantly altered s-100*β* protein in the two depression subgroups, providing a valuable biological marker to identify UD and BD.

### 4.3. An Effective Prediction Model Based on Network, Cytokines, and Anxiety Symptom

Instead of using imaging or biological markers separately to distinguish uni- and bipolar depression in previous studies [14, 24, 45, 46], we combined depressive and anxiety symptoms, inflammatory and growth factors, and brain network imaging markers to predict risk factors for UD and BD. Innovatively, the unipolar vs. bipolar depression classification model achieved a high accuracy rate with six biomarkers (IL-8, IL-17, s-100*β*, the nodal efficiency of the right amygdala, the prefrontal-cingulate-amygdala subnetwork intensity, and anxiety symptoms). Meanwhile, it suggested that increased levels of IL-8, IL-17, and s-100*β* levels, raised nodal efficiency of the right amygdala, and elevated intensity of the prefrontal-cingulate-amygdala subnetwork were risk factors for diagnosing UD. In contrast, high anxiety symptoms tended to closely correlate with BD. In line with previous studies, this study also showed that anxiety disorders could be a risk factor for bipolar disorders [47], with significantly higher rates of comorbid anxiety disorders in BD than in UD patients [48].

Substantial evidence suggested that disrupted prefrontal-amygdala functional connectivity could be a potential biomarker for UD and BD [49–53]. As a complement, we provided structural evidence of differences between the patient groups and the HC group. Relative to healthy controls, unipolar patients showed increased global and local efficiency but decreased the length of the shortest path, suggesting an overused structural network in the emotion circuit, particularly in the right amygdala and prefrontal orbital areas, which presents as increased nodal efficiency in the right anterior cingulate gyrus in bipolar patients.

There were also some limitations in this study. First, the sample sizes for unipolar and bipolar depression are relatively small, so the findings cannot be replicated in an independent data set. Second, although we considered biological samples, imaging data, and clinical scales to distinguish the two types of depression, these factors are still insufficient to distinguish and elucidate the potential mechanisms of emotion processing in the two disorders. Future studies need to incorporate additional clinical factors such as sex, duration of illness, number of episodes, and genetic factors to develop a more comprehensive diagnostic kit that can be used to guide clinical therapy.

## 5. Conclusions

This study identified biological and neurobiological markers to help differentiate unipolar and bipolar depression. In all, significant risk factors, e.g., s-100*β* protein, amygdala nodal efficiency, and prefrontal cingulate-amygdala subnetwork intensity, showed statistical differences between UD and BD. Importantly, combining IL-8, IL-17, and anxiety symptoms, we found that these six risk factors can accurately distinguish two different depressions. These findings fill a gap in the brain-wide structural network of emotional circuits in bipolar and unipolar depression, pointing to different emotion-processing mechanisms in both disorders.

## Figures and Tables

**Table 1:** Comparison of demographic, psychological, and clinical information between groups.

	UD (*n* = 37)	BD (*n* = 31)	HC (*n* = 61)	*F/t/χ* ^2^	*p* value
Age (years)	25.68 ± 3.78	23.32 ± 6.21	25.36 ± 4.28	2.54	0.08
Sex (male/female)^1^	16/21	10/21	27/34	1.32	0.52
Education (years)	14.76 ± 3.59	14.23 ± 2.62	13.95 ± 3.50	0.67	0.51
Marital status^1^				0.69	0.95
Married/unmarried/	6/30/1/0	3/26/1/0	7/51/2/0		
Divorced/widowhood					
Monthly income (¥)^1^				3.82	0.70
>5000	14	6	24		
3000-5000	12	7	21		
1000-3000	4	4	14		
<1000	2	0	1		
Smoking status^1^				4.97	0.08
None	31	20	56		
Smoking	5	7	5		
Course of disease (months)^2^	13.41 ± 17.51	34.35 ± 33.79	N/A	2.42	0.02⁣^∗^
Comorbid mental illness	No.	Anxiety disorder (2)	N/A		
Somatic disease	Duodenal ulcer (1) Gastric ulcer (1) Mastadenoma (1)	No.	No.		
Unmedicated (%)	83.8%	83.9%	N/A		
Current medication	Duloxetine (1) Milnacipran and sulpiride (3) Quetiapine (1) Escitalopram (1)	Lithium (1) Lurasidone (1) Valproic acid (1) Lamotrigine (1) Olanzapine and sertraline (1)	N/A		
HAM-D	17.81 ± 6.00	19.70 ± 4.32	1.38 ± 1.58	301.24	<0.001⁣^∗∗∗^
HAM-A	16.30 ± 6.40	18.40 ± 4.90	1.07 ± 1.41	229.68	<0.001⁣^∗∗∗^

Note: ^1^chi-square test. ^2^Two independent-sample *t*-tests (two-tailed). Other analyses were ANOVA with Bonferroni's post hoc test. Descriptive statistics for continuous variables showed as mean ± standard deviation (SD). ⁣^∗^*p* < 0.05 and ⁣^∗∗∗^*p* < 0.001. UD: unipolar depression; BD: bipolar depression; HC: healthy controls; HAM-D: Hamilton Depression Rating Scale; HAM-A: Hamilton Anxiety Rating Scale; N/A: not applicable.

**Table 2:** Comparison of graph properties of the prefrontal-limbic structural network.

	UD (*n* = 37)	BD (*n* = 31)	HC (*n* = 61)	*F*	*p* value	Post hoc tests
Global network properties						
*E*_glob_ (×10^−2^)	11.34 ± 0.71	11.00 ± 0.69	10.56 ± 1.40	5.99	0.003	UD vs. HC, *p* = 0.003
*E*_loc_ (×10^−2^)	13.46 ± 0.89	13.21 ± 0.93	12.65 ± 1.57	5.23	0.007	UD vs. HC, *p* = 0.008
*L*_*p*_	1.99 ± 0.15	2.04 ± 0.14	2.17 ± 0.35	5.62	0.005	UD vs. HC, *p* = 0.005
Nodal network properties (×10^−2^)						
Nodal efficiency						
ORBsup.R	14.27 ± 1.08	13.57 ± 1.06	13.08 ± 1.81	7.72	0.007	UD vs. HC, *p* = 0.001
ORBmed.R	12.81 ± 1.67	11.91 ± 1.51	11.41 ± 2.04	7.09	0.001	UD vs. HC, *p* = 0.001
OLF.L	11.38 ± 1.10	10.57 ± 1.43	10.17 ± 1.96	6.35	0.002	UD vs. HC, *p* = 0.002
ACG.R	13.95 ± 1.04	13.79 ± 1.13	12.88 ± 1.91	6.79	0.002	UD vs. HC, *p* = 0.003 BD vs. HC, *p* = 0.023
Amygdala.R	7.60 ± 1.28	6.84 ± 1.27	6.37 ± 1.53	9.00	0.0002	UD vs. HC, *p* < 0.0001 UD vs. BD, *p* = 0.016
TPOsup.R	10.54 ± 1.60	10.21 ± 1.62	9.46 ± 1.70	5.51	0.005	UD vs. HC, *p* = 0.007
Nodal local efficiency						
MCG.R	18.88 ± 3.55	17.47 ± 3.67	15.68 ± 5.10	6.60	0.002	UD vs. HC, *p* = 0.002

Note: the reported metrics are represented using the fitted area under curve (AUC) values of the graph-theoretical properties spanned by different network sparsity values. The significance of network metrics was corrected by FDR, with age and sex as covariates. Bonferroni's correction (*p* < 0.05) was used for post hoc tests. Abbreviations: UD: unipolar depression; BD: bipolar depression; HC: healthy controls; *E*_glob_: global efficiency; *E*_loc_: local efficiency; *L*_*p*_: shortest path length; ORBsup: superior frontal gyrus, orbital part; ORBmed: medial frontal gyrus, orbital part; OLF: olfactory cortex; ACG: anterior cingulate and paracingulate gyri; TPOsup: temporal pole, superior temporal gyrus; MCG: medial cingulate and paracingulate gyri; L: left; R: right.

**Table 3:** Logistic regression showing odd rations of predictors differentiating patients with UD from patients with BD.

Variables	Coefficient *B*	SEM	*z*	Wald *χ*^2^	*p* value	OR	OR 95% CI		AUC
							Lower	Upper	
s-100*β*	0.017	0.008	2.226	4.956	0.026	1.017	1.002	1.033	0.685
IL-8	0.063	0.026	2.428	5.894	0.015	1.064	1.012	1.120	0.665
IL-12	0.133	0.069	1.914	3.662	0.056	1.142	0.997	1.308	0.506
IL-17	0.268	0.131	2.041	4.167	0.041	1.307	1.011	1.691	0.598
HAM-A	-0.296	0.134	-2.211	4.888	0.027	0.744	0.572	0.967	0.373
The intensity of the prefrontal-cingulate-amygdala subnetwork	0.181	0.075	2.422	5.867	0.015	1.198	1.035	1.387	0.732
The right amygdala efficiency	0.205	0.079	2.579	6.651	0.010	1.227	1.050	1.434	0.753
The prediction model^a^									0.949

Note: UD: unipolar depression; BD: bipolar depression; OR: odds ratio; CI: confidence interval; SEM: standard error of the mean; HAM-A: Hamilton Anxiety Rating Scale; AUC: area under curve. ^a^Sensitivity 0.875; specificity 0.938.

## Data Availability

The data that support the findings of this study are available from the corresponding authors upon reasonable request.

## Supplementary Materials

Supplementary Materials Supplemental Table 1 presents detailed definitions and abbreviations of the nodes in the emotion circuit, which refer to atlas AAL90. Supplemental Table 2 displays detailed statistics on the comparisons of plasma inflammatory mediators and growth factors among the UD, BD, and HC groups. Supplemental Table 3 provides a detailed analysis of the between-group differences in the components of subnetworks in the UD, BD, and HC groups. The supplemental materials contain a detailed explanation of the network properties.
