# Supplementary material for: Characterizing Unipolar and Bipolar Depression by Alterations in Inflammatory Mediators and the Prefrontal-Limbic Structural Network
Source: Depress Anxiety. 2023 May 24;2023:5522658. doi: 10.1155/2023/5522658 (PMC11921842; doi:10.1155/2023/5522658)
Supplement: Supplementary Materials — Supplemental Table 1 presents detailed definitions and abbreviations of the nodes in the emotion circuit, which refer to atlas AAL90. Supplemental Table 2 displays detailed statistics on the comparisons of plasma inflammatory mediators and growth factors among the UD, BD, and HC groups. Supplemental Table 3 provides a detailed analysis of the between-group differences in the components of subnetworks in the UD, BD, and HC groups. The supplemental materials contain a detailed explanation of the network properties. [file 5522658.f1.docx]

| **Supplemental Table 1.** Network nodes in the prefrontal-limbic system based on the automated anatomical labeling (AAL) atlas | | | | | |
| --- | --- | --- | --- | --- | --- |
| AAL Index | Region | Abbreviations | AAL Index | Region | Abbreviations |
| (5,6) | Superior frontal gyrus, orbital part | ORBsup | (33,34) | Median cingulate and paracingulate gyri | MCG |
| (9, 10) | Middle frontal gyrus, orbital part | ORBmid | (35,36) | Posterior cingulate gyrus | PCG |
| (15,16) | Inferior frontal gyrus, orbital part | ORBinf | (37,38) | Hippocampus | HIP |
| (21,22) | Olfactory cortex | OLF | (39,40) | Parahippocampal gyrus | PHG |
| (25,26) | Medial frontal gyrus, orbital part | ORBmed | (41,42) | Amygdala | Amygdala |
| (27,28) | Gyrus rectus | REC | (77,78) | Thalamus | THA |
| (29,30) | Insula | INS | (83,84) | Temporal pole: superior temporal gyrus | TPOsup |
| (31,32) | Anterior cingulate and paracingulate gyri | ACG | (87,88) | Temporal pole: middle temporal gyrus | TPOmid |

| **Supplemental Table 2.** Levels of plasma cytokines in three groups | | | | | | |
| --- | --- | --- | --- | --- | --- | --- |
|  | UD  (n=34) | BD  (n=17) | HC  (n=55) | ANOVA  (*F value*) | *P*  *value* | Two sample t-test |
| CRP (mg/L) | 11.16 ± 2.68 | 11.25 ± 2.50 | 11.36 ± 2.33 | 0.08 | 0.93 |  |
| IL_1β (pg/mL) | 21.01 ± 5.01 | 20.08 ± 4.94 | 20.65 ± 4.59 | 0.21 | 0.81 |  |
| IL_2 (pg/mL) | 290.68 ± 61.14 | 299.17 ± 58.09 | 299.04 ± 62.60 | 0.22 | 0.81 |  |
| IL_4 (pg/mL) | 43.93 ± 10.95 | 47.73 ± 11.88 | 44.31 ± 11.11 | 0.74 | 0.48 |  |
| IL_6 (pg/mL) | 121.13 ± 22.70 | 116.19 ± 28.19 | 118.01 ± 25.44 | 0.27 | 0.77 |  |
| IL_8 (pg/mL) | 173.38 ± 36.10 | 156.63 ± 28.17 | 168.56 ± 35.86 | 1.32 | 0.27 |  |
| IL_10 (pg/mL) | 21.11 ± 6.13 | 19.56 ± 5.53 | 20.72 ± 5.36 | 0.43 | 0.65 |  |
| IL_12 (pg/mL) | 39.38 ± 9.96 | 39.67 ± 10.69 | 39.56 ± 10.57 | 0.01 | 0.99 |  |
| IL_17 (pg/mL) | 34.49 ± 8.39 | 33.19 ± 8.13 | 33.99 ± 6.92 | 0.17 | 0.85 |  |
| s-100β (pg/mL) | 441.96 ± 123.92 | 370.28 ± 110.73 | 427.67 ± 124.32 | 2.03 | 0.14 | UD vs. BD,  *t* = 2.02, *p* = 0.049* |
| IFN_γ (pg/mL) | 977.49 ± 193.48 | 957.36 ± 197.31 | 925.90 ± 174.78 | 0.85 | 0.43 |  |
| TNF_α (pg/mL) | 113.33 ± 20.17 | 113.46 ± 19.19 | 109.06 ± 18.85 | 0.66 | 0.52 |  |
| BDNF (pg/mL) | 901.86 ± 220.34 | 914.46 ± 223.41 | 906.29 ± 219.51 | 0.02 | 0.98 |  |
| GDNF (pg/mL) | 1066.85 ± 223.04 | 1050.25 ± 173.83 | 1065.37 ±216.93 | 0.04 | 0.96 |  |
| Note. UD, unipolar depression. BD, bipolar depression. HC, Healthy Controls. ANOVA, analysis of variance. CRP, C-reactive protein. IL, Interleukin. IFN-γ, Interferon-gamma. TNF-α, Tumor Necrosis Factor-α. BDNF, Brain-Derived Neurotrophic Factor. GDNF, Glial Cell-Derived Neurotrophic Factor. * *p* < 0.05. | | | | | | |

| **Supplemental Table 3.** Between-group differences of subnetworks in the prefrontal-limbic system in NBS analyses | | | | | |
| --- | --- | --- | --- | --- | --- |
| **Components** | **Prefrontal subnetwork** | **Test statistic**  **(t values)** |  | **Prefrontal-cingulate-amygdala subnetwork** | **Test statistic**  **(*t* values)** |
| UD > HC | ORBmid.R to ORBsup.R | 3.17 | UD > BD | ORBmed.R to ORBsup.R | 3.11 |
|  | ORBmed.L to ORBsup.R | 3.04 |  | ORBmed.R to Olfactory.R | 2.22 |
|  | ORBmed.R to ORBsup.R | 2.68 |  | Amygdala.R to Olfactory.R. | 2.54 |
|  | Rectus.L to ORBsup.R | 2.53 |  | ORBmed.R to ACG.R | 2.07 |
|  |  |  |  | MCG.L to ACG.R | 3.23 |
|  |  |  |  | MCG.R to ACG.R | 2.05 |
|  |  |  |  | MCG.L to PCG.R | 2.65 |
| Note. UD, unipolar depression. BD, bipolar depression. HC, Healthy Controls. ORBmid.R, right middle frontal gyrus, orbital part. ORBsup.R, right superior frontal gyrus, orbital part. ORBmed.L, left medial frontal gyrus, orbital part. Rectus.L, left olfactory cortex. ACG.R, right anterior cingulate and paracingulate gyri. MCG.L, left median cingulate and paracingulate gyri. MCG.R, median cingulate and paracingulate gyri. PCG.R, posterior cingulate gyrus. The first right column means that the statistical values of the t-test represent the structural connectivity differences between groups. | | | | | |

**Network properties**

***Shortest path length (L_p_)*** The shortest path length, L_p_, is the average shortest path length between all possible pairs of nodes in a network, where *l_i_* is the average shortest path length from node *i* to all other nodes.

$$\boldsymbol{L}_{\boldsymbol{p}}\boldsymbol{=}\frac{\boldsymbol{1}}{\boldsymbol{N}}\sum_{\boldsymbol{i}} \boldsymbol{l}_{\boldsymbol{i}}$$

***Network efficiency*** The global efficiency, ***E_glob_***, is an alternative measure of integration that is closely related to the characteristic path length, which measures the global efficiency of the parallel information transfer in the network (1). The global efficiency of a network is the reciprocal of the harmonic mean of its path lengths, where ***l_ij_*** is the shortest path length between node ***i*** and node ***j***.

$$\boldsymbol{E}_{\boldsymbol{glob}}\boldsymbol{=}\frac{\boldsymbol{1}}{\boldsymbol{N(N-1)}}\sum_{\boldsymbol{i\neq j}} \frac{\boldsymbol{1}}{\boldsymbol{l}_{\boldsymbol{ij}}}$$

Latora and Marchiori (2001) also defined a node-specific measure known as the local efficiency. They define local efficiency of the ***I*** node, E_loc_ (***i***), such that

$$\boldsymbol{E}_{\boldsymbol{loc}}\left( \boldsymbol{i} \right)\boldsymbol{=}\frac{\boldsymbol{1}}{\boldsymbol{N}_{\boldsymbol{Gi}}\left( \boldsymbol{N}_{\boldsymbol{Gi}}\boldsymbol{-1} \right)}\sum_{\boldsymbol{j, h \in Gi}} \frac{\boldsymbol{1}}{\boldsymbol{l}_{\boldsymbol{jh}}}\boldsymbol{,}$$

where ***G_i_*** denotes the subgraph comprising all nodes that are immediate neighbors of the ***i***th node. In other words, the local efficiency of node ***i*** is defined with respect to the subgraph comprising all neighbors of *i,* after removal of ***i*** and its incident edges. The local efficiencies for each node can be averaged over all nodes to estimate the mean local efficiency of a network.

The node efficiency is the normalized sum of the reciprocal of the shortest path lengths from a given node to all other nodes in the network (2). The measure is used to localize global efficiency effects to specific nodes. Nodal efficiency quantifies how well a given region is integrated within the network via its shortest paths. Specifically, the nodal, or regional, efficiency of node ***j*** is given by, where ***l_ij_*** is the shortest path length between node ***i*** and node ***j***.

$$\boldsymbol{E}_{\boldsymbol{nodal}}\left( \boldsymbol{j} \right)\boldsymbol{=}\frac{\boldsymbol{1}}{\boldsymbol{N-1}}\sum_{\boldsymbol{i}} \frac{\boldsymbol{1}}{\boldsymbol{l}_{\boldsymbol{ij}}}$$

**References**

1. Latora V, Marchiori M. Efficient behavior of small-world networks. Phys Rev Lett. 2001 Nov 5;87(19):198701.

2. Achard S, Bullmore E. Efficiency and cost of economical brain functional networks. PLoS Comput Biol. 2007 Feb 2;3(2): e17.
